# A case report of concurrent management of acute myocardial infarction complicated by left ventricular thrombus and ischaemic stroke

**DOI:** 10.1093/ehjcr/ytae193

**Published:** 2024-04-15

**Authors:** Yuka Kodama, Kenji Matsumoto, Hisashi Kubota, Onichi Furuya, Yoshio Kawase

**Affiliations:** Department of Cardiology, Izumi City General Hospital, 4-5-1 Wake-cho, Izumi 594-0073, Japan; Department of Cardiology, Izumi City General Hospital, 4-5-1 Wake-cho, Izumi 594-0073, Japan; Department of Neurosurgery, Kubota Clinic Neurosurgery, Izumi, Japan; Department of Cardiovascular Surgery, Kishiwada Tokushukai Hospital, Kishiwada, Japan; Department of Cardiology, Izumi City General Hospital, 4-5-1 Wake-cho, Izumi 594-0073, Japan

**Keywords:** Myocardial infarction, Stroke, Left ventricular thrombus, Heart–brain team approach, Case report

## Abstract

**Background:**

Left ventricular thrombus (LVT) formation is a serious complication of acute myocardial infarction (AMI) requiring complicated management strategies and collaboration among cardiologists, cardiovascular surgeons, and neurosurgeons.

**Case summary:**

We present the case of an 83-year-old female patient with AMI. Emergency coronary angiography revealed subtotal occlusion of the proximal left anterior descending artery, and the patient was successfully treated with a drug-eluting stent. The following day, she suddenly developed loss of consciousness, global aphasia, and right hemiplegia. Brain magnetic resonance imaging revealed acute ischaemic cerebral infarction caused by multiple mobile LVT, as demonstrated by echocardiography. After a heart–brain team discussion, we decided to perform percutaneous mechanical thrombectomy. Successful recanalization was achieved with mechanical thrombectomy 2 h after presentation, which resulted in significant neurological recovery. Immediately after the thrombectomy, she was transferred to a cardiovascular surgery centre for surgical removal of multiple LV apical thrombi. Two weeks after the operation, the patient was discharged with the recovery of LV systolic function.

**Discussion:**

Although AMI complicated by acute stroke caused by LVT remains a clinical challenge, a multidisciplinary approach is critically important for optimal care. Based on an urgent team discussion, we decided to perform endovascular thrombectomy for ischaemic stroke, followed by surgical removal of the LVT, requiring patient transportation to the cardiovascular surgery centre. Given that the heart and brain team-based approach remains confined to large, specialized centres, it might be beneficial to establish a community-based integrated heart–brain team that can address the growing needs of complex patients.

**Learning points**

- Left ventricular thrombus (LVT) following acute myocardial infarction (AMI), which can lead to stroke, remains a matter of concern. As shown in the present case with LVT observed despite the preventive use of anticoagulants, early diagnosis is vital to avoid subsequent thromboembolism.
- For AMI complicated by acute stroke caused by LVT, a multidisciplinary team approach is critically important for the optimal care. Given that the heart and brain team-based approach remains confined to large, specialized centres, it might be beneficial to establish a community-based integrated heart–brain team that can address the growing needs of complex patients.

## Introduction

Left ventricular thrombus (LVT) formation is a serious complication of acute myocardial infarction (AMI) that may cause severe complications such as stroke or peripheral embolism.^1^ Cerebral embolism events are among the most critical complications in the acute phase and requires complicated management strategies with collaboration among cardiologists, cardiovascular surgeons, and neurosurgeons. The incidence of LVT after AMI has significantly declined since the introduction of reperfusion therapies; however, LVT is still present in 3.9% of patients with new-onset anterior AMI in the era of primary percutaneous coronary intervention (PCI) and potent dual antiplatelet therapy (DAPT).^2,3^ Moreover, an effective strategy to prevent post-MI LVT remains a clinical challenge.

## Summary figure

**Figure:**
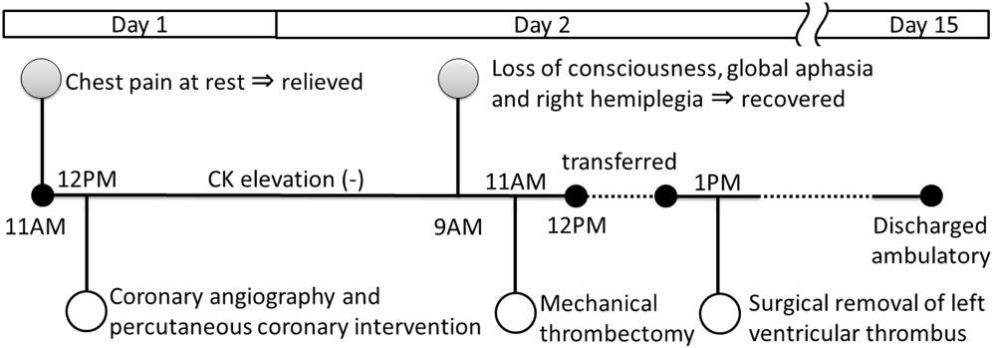


## Case presentation

An 83-year-old female presented to her family physician with chest pain that started 5 days before admission. The patient was later referred to our hospital because neither electrocardiography nor blood tests revealed abnormal findings. Although the symptoms resolved spontaneously, the patient suddenly complained of persistent chest pain in the examination room of our hospital. As the electrocardiogram revealed ST-segment elevation, the patient was transported to the emergency room. She had no notable medical history. Her current medication consisted of rabeprazole 10 mg once daily for reflux oesophagitis, and minodronate 50 mg once every 4 weeks and eldecalcitol 0.75 μg once daily for osteoporosis. Vital signs showed no abnormalities except for the heart rate, which was 104 beats per minute. She had a third heart sound, but other physical examination results were normal.

**Figure 1:**
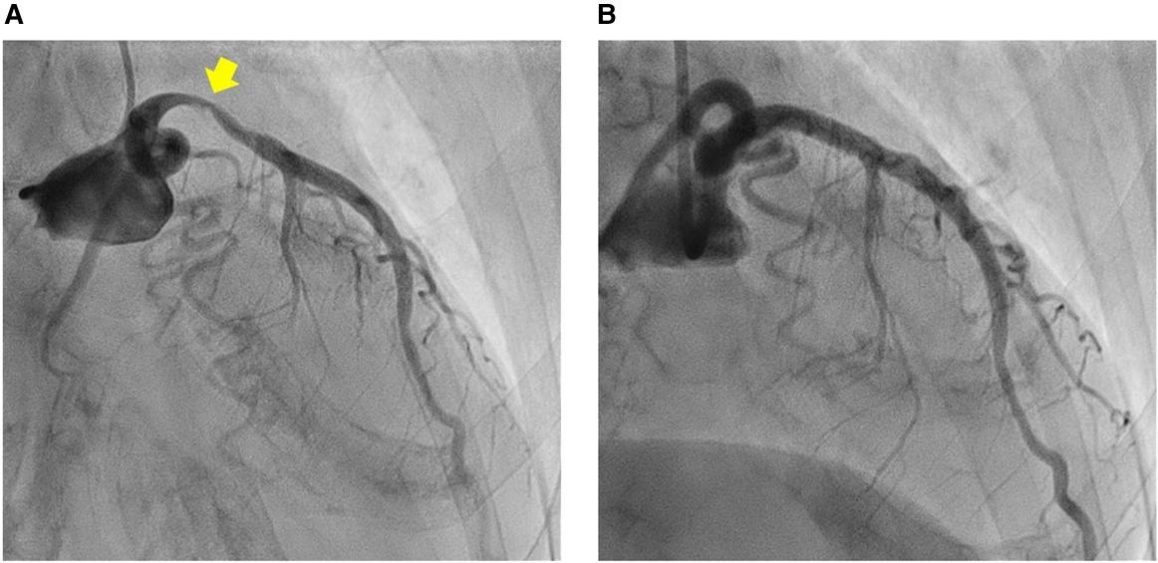
(*A*) Coronary angiography demonstrated a subtotal occlusion of the proximal left anterior descending artery. (*B*) Final angiography showed successful revascularization with a drug-eluting stent implantation.

Electrocardiography (see Supplementary material online, *Figure S1*) revealed ST-segment elevation in leads I, aVL, and from V2 to V6, indicative of acute ST-elevation MI (STEMI). Blood tests (see Supplementary material online, *Table S1*) showed a troponin-I level of 3.08 ng/mL (normal range, <0.05), and creatine kinase (CK)-MB level of 18 IU/L (normal range, 4–16). Furthermore, echocardiography revealed severely decreased LV function (ejection fraction 30%) with akinetic movement of the LV anterior wall and apex. An emergency coronary angiography was performed. Coronary angiography revealed subtotal occlusion of the proximal left anterior descending artery (*Figure 1A*). After drug-eluting stent implantation, the final angiography showed optimal results (*Figure 1B*). Her chest pain was relieved soon after PCI. The patient was moved to the intensive care unit with continuous intravenous heparin to prevent re-infarction and thromboembolism. She was administered aspirin 100 mg once daily, clopidogrel 75 mg once daily, bisoprolol 1.25 mg once daily, and rosuvastatin 2.5 mg once daily. During follow-up, the CK levels were not significantly elevated.

**Figure 2:**
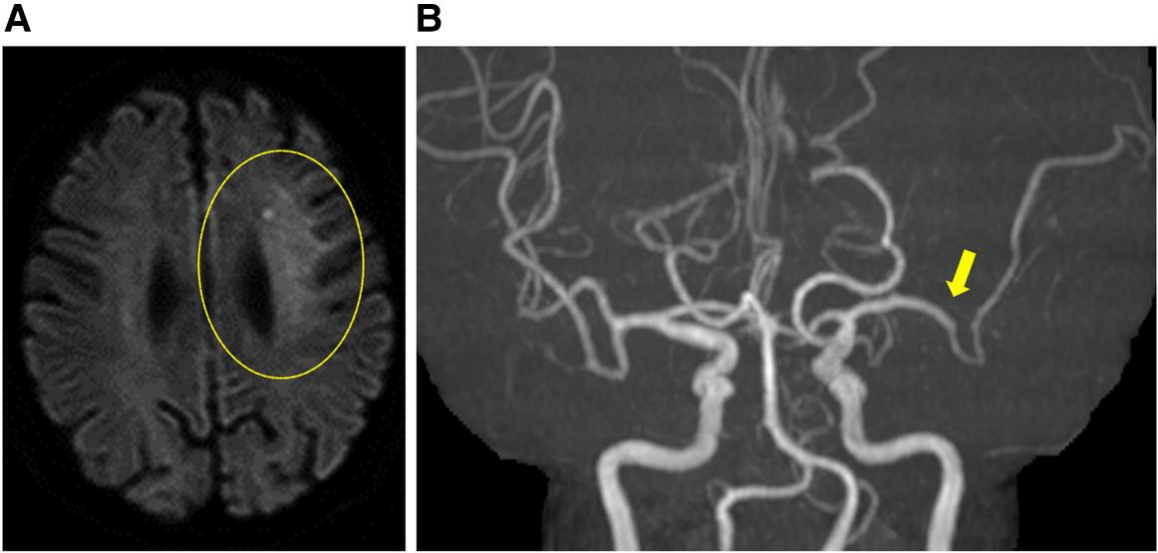
(*A*) Brain magnetic resonance imaging with diffusion-weighted imaging showed an acute infarction in the left frontal lobe (circle). (*B*) Magnetic resonance angiography showed an occlusion of the anterior branch of the left middle cerebral artery (arrow).

**Figure 3:**
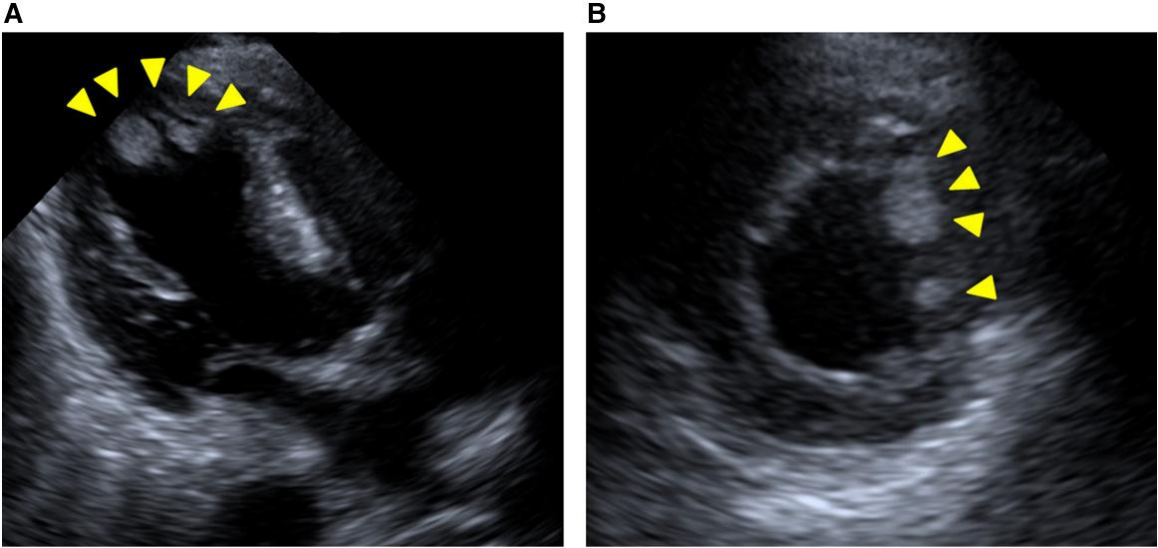
Transthoracic echocardiography showed multiple mobile thrombi in the apex of the left ventricle (arrowheads). (*A*) Apical three-chamber view. (*B*) Parasternal-short axis view at the apex.

The following day, the patient suddenly developed a loss of consciousness, global aphasia, and right hemiplegia, with a National Institute of Health Stroke Scale of 19. She was last seen normal 30 min before the presentation. Brain magnetic resonance (MR) imaging revealed acute ischaemic cerebral infarction in the left frontal lobe (*Figure 2A*), and MR angiography revealed occlusion of the anterior branch of the left middle cerebral artery (*Figure 2B*). Echocardiography revealed mobile and protruding apical thrombi in the left ventricle (10 × 13 mm, 6 × 10 mm), which were considered the embolic source (*Figure 3*). We held an urgent team meeting with cardiologists, cardiovascular surgeons, and neurosurgeons to discuss the management of these complications after AMI. The patient received DAPT and modified therapeutic heparin, which is contraindicated for tissue plasminogen activators. Therefore, endovascular therapy with mechanical thrombectomy was considered the optimal treatment option for acute ischaemic stroke. In addition, given that large LVTs are highly mobile and protruding, the patient should be referred for surgical thrombectomy. Finally, we decided to perform mechanical thrombectomy, followed by surgical removal of the LVT.

**Figure 4:**
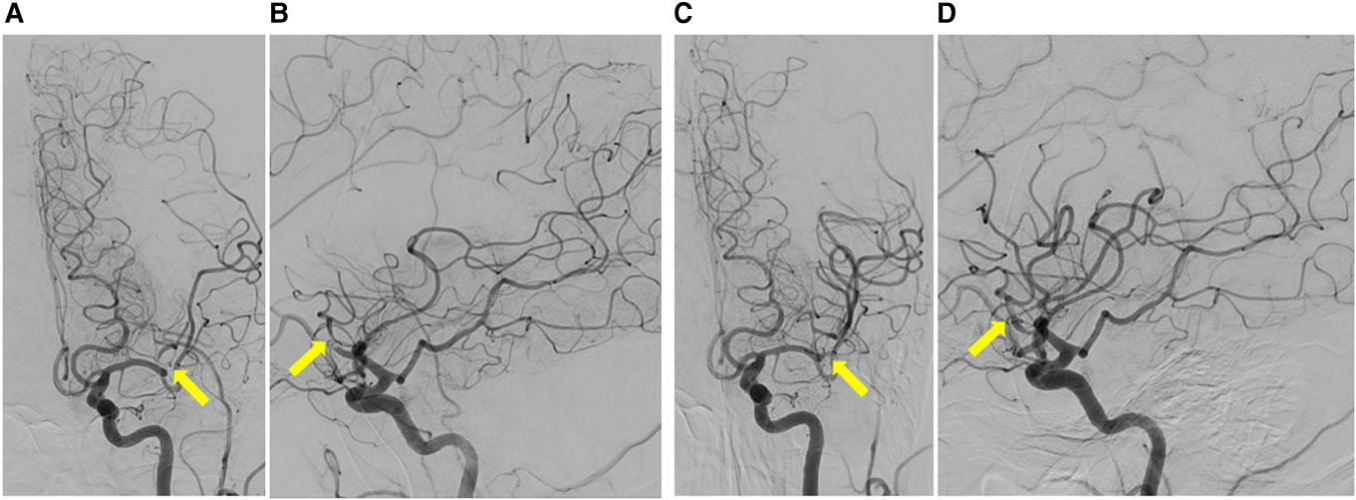
Digital subtraction angiography with anteroposterior (*A*) and lateral (*B*) views demonstrated occlusion of the superior trunk of the anterior branch of the left middle cerebral artery (arrows). (*C* and *D*) Post-thrombectomy angiography showed complete recanalization of the target vessel (arrows).

**Figure 5:**
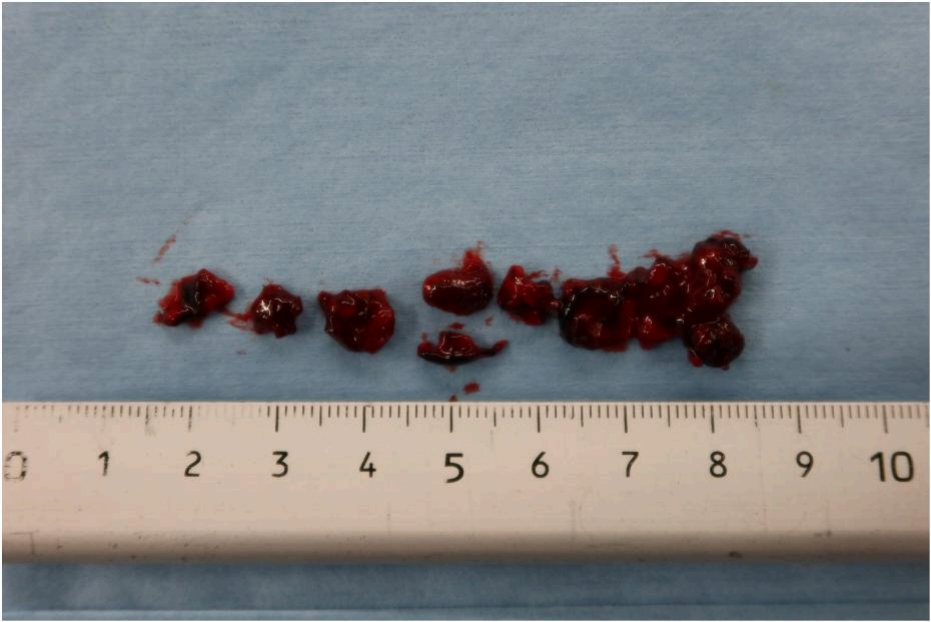
Left ventricular thrombi removed from left ventricular apex.

Two hours after symptom onset, successful recanalization was achieved with mechanical thrombectomy using a stent retriever, which resulted in significant neurological recovery (*Figure 4*). Immediately after the thrombectomy, the patient was transferred to a cardiovascular surgery centre because open-heart surgery was not available in our hospital. Twenty-six hours after the AMI onset, the patient underwent surgical removal via a left ventriculotomy without ventricular restoration. Multiple thrombi were removed from the LV apex (*Figure 5*). Two weeks after the operation, the patient was discharged with the recovery of LV systolic function. Warfarin was initiated in addition to DAPT to prevent the recurrence of LVT; clopidogrel was stopped three weeks later due to the high bleeding risk (HBR), particularly underweight (body mass index of 15 kg/m²).

Two months after the onset of AMI and stroke, complementary workup investigations for thrombophilia did not show thrombotic disorders such as deficiency of proteins C and S, elevated factor VIII and IX, antiphospholipid syndrome, or heparin-induced thrombocytopaenia.

## Discussion

Here, we present a case in which AMI, stroke, and LVT were concurrently managed through an interactive team discussion.

The 2013 American College of Cardiology/American Heart Association guidelines provide a Class IIb indication for prophylactic anticoagulation among patients with STEMI and anterior apical akinesis or dyskinesis.^4^ Since our patient was deemed to be at very high risk for LVT including low ejection fraction and severe antero-apical wall motion abnormality,^1,3,5^ we administered intravenous heparin to prevent LVT after PCI.^6^ Considering that LVT might be observed despite the preventive use of anticoagulants, early diagnosis is vital to avoid subsequent thromboembolism.

Decisions concerning LVT treatment remain challenging. Although surgical removal of LVT is an option for patients with a high-embolic risk profile, its procedural risks are often considered higher than the benefits.^7^ Indeed, there are only a few anecdotal reports and retrospective small case series of surgical excision of LVT.^7–9^ The concern with surgical removal of thrombus is the risk of LV ventriculotomy in a friable myocardium with an increased risk of cardiogenic shock, arrhythmia, myocardial necrosis, and haemorrhage.^8^ However, we observed no increase in CK after PCI, which indicated that myocardial injury was minimized. Considering the risk factors for stroke recurrence, including the multiple, protuberant, and highly mobile nature of the LVT and the onset of acute stroke despite anticoagulation,^2,4,10^ LV ventriculotomy could be a better treatment option than anticoagulation for preventing stroke recurrence. Therefore, surgical removal via a left ventriculotomy was performed. According to the current Japanese Circulation Society guidelines,^11^ triple therapy with warfarin and DAPT was administered for the first three weeks after surgery considering the patient’s HBR. With limited data for optimal management for preventing recurrent embolism, the duration and combination of the antithrombotic therapy should be carefully determined using a case-by-case approach.^2,4,12^

A heart and brain team plays an important role in the management of various cardiovascular and neurological disorders, which enables the selection of well-balanced therapeutic options.^13^ According to the urgent team discussion, we decided on endovascular thrombectomy for ischaemic stroke followed by surgical removal of LVT requiring patient transportation to the cardiovascular surgery centre. Nevertheless, the patient recovered significantly without serious adverse events. Since the heart and brain team-based approach remains confined to large, specialized centres, it might be beneficial to establish a community-based integrated heart–brain team that can address the growing needs of complex patients.

## Supplementary Material

ytae193_Supplementary_Data

## Data Availability

The data underlying this article will be shared upon reasonable request to the corresponding author.

## Lead author biography

Dr Yuka Kodama has graduated from medical school in the Osaka Metropolitan University. Upon completion of the junior residency for two years, she is currently a senior resident in internal medicine and cardiology at the Izumi City General Hospital in Japan. She has a special interest in coronary haemodynamics and echocardiographic imaging.
