# Supplementary material for: A case report of concurrent management of acute myocardial infarction complicated by left ventricular thrombus and ischaemic stroke
Source: Eur Heart J Case Rep. 2024 Apr 15;8(4):ytae193. doi: 10.1093/ehjcr/ytae193 (PMC11050652; doi:10.1093/ehjcr/ytae193)
Supplement: ytae193_Supplementary_Data [file ytae193_supplementary_data.doc]

**Supplementary Table S1 Baseline laboratory data**

|  | Values | Normal ranges |
| --- | --- | --- |
| White blood cell count (/μL) | 8100 | 3300–8600 |
| Neutrophil (%) | 67.6 | 40.0–70.0 |
| Lymphocyte (%) | 24 | 22.0–56.0 |
| Monocyte (%) | 7.6 | 2.0–12.0 |
| Eosinophil (%) | 0.5 | 0–7 |
| Basophil (%) | 0.3 | 0–3.0 |
| Haemoglobin (g/dL) | 14.1 | 11.6–14.8 |
| Haematocrit (%) | 44.4 | 35.1–44.4 |
| Platelet (x10^4^/μL) | 37.6 | 15.8–34.8 |
| Total protein (g/dL) | 6.4 | 6.6–8.1 |
| Albumin (g/dL) | 3.7 | 4.1–5.1 |
| Total bilirubin (mg/dL) | 0.71 | 0.4–1.5 |
| Aspartate aminotransferase (IU/L) | 33 | 13–30 |
| Alanine aminotransferase (IU/L) | 13 | 7–23 |
| Lactate dehydrogenase (IU/L) | 254 | 124–222 |
| Alkaline phosphatase (IU/L) | 55 | 38–113 |
| γ-glutamyl transpeptidase (IU/L) | 19 | 9–32 |
| Creatine kinase (IU/L) | 107 | 41–153 |
| Creatine kinase-myocardial band (IU/L) | 18 | 4–16 |
| Total cholesterol (mg/dL) | 174 | 142–248 |
| High-density lipoprotein cholesterol (mg/dL) | 61 | 48–103 |
| Low-density lipoprotein cholesterol (mg/dL) | 86 | 65–163 |
| Triglyceride (mg/dL) | 91 | 30–117 |
| Blood urea nitrogen (mg/dL) | 18.9 | 8.0–20.0 |
| Creatinine (mg/dL) | 0.73 | 0.46–0.79 |
| estimated glomerular filtration rate (mL/min/1.73 m^2^) | 57 |  |
| Uric acid (mg/dL) | 4.8 | 2.6–5.5 |
| Serum sodium (mEq/L) | 138 | 138–145 |
| Serum potassium (mEq/L) | 4.4 | 3.6–4.8 |
| C-reactive protein (mg/dL) | 0.85 | <0.14 |
| Brain natriuretic peptide (pg/mL) | 527.5 | <18.4 |
| Troponin-I (ng/mL) | 3.08 | <0.05 |
| Activated partial thromboplastin time (sec) | 23.1 | 24.0–34.0 |
| Prothrombin time-international normalized ratio | 1.03 | 0.90–1.10 |
| Fibrin degradation product (μg/mL) | 4.7 | <5.0 |
| D-dimer (μg/mL) | 2.2 | <1.0 |


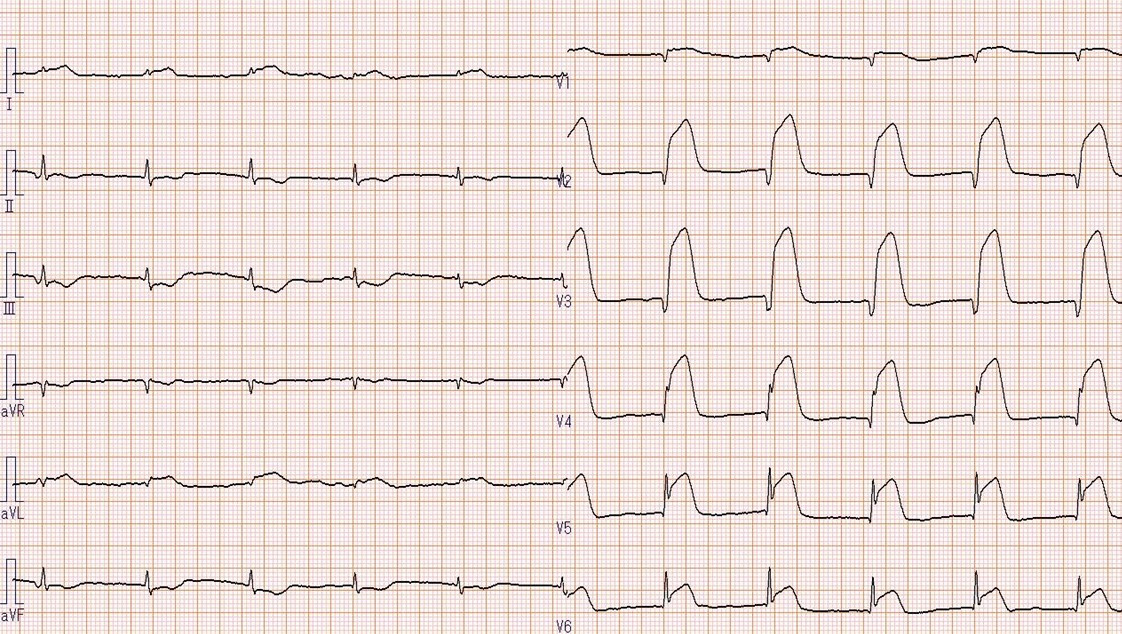


**Supplementary Figure S1 Electrocardiogram**
